# Carcinoma of the colon in a 40 year old female: a case report

**DOI:** 10.11604/pamj.2019.33.161.12383

**Published:** 2019-07-03

**Authors:** Jonathan Walubembe, Mike Engola Aremo, Conrad Tumwine, Florence Nabaale, Andrew Twinomasiko, Isaac Tukesiga, Phillip Ssenyomo, Apollo Mugaiga, Jerome Roy Semakula, Derrick Amone Olwedo, David Lagoro Kitara

**Affiliations:** 1 Fifth year Medical Student, Department of Surgery, Faculty of Medicine, Gulu University, Gulu, Uganda; 2 Gulu Regional Referral Hospital, Ministry of Health, Gulu, Uganda; 3 Department of Surgery, Faculty of Medicine, Gulu University, Gulu, Uganda; 4 Department of Surgery, Faculty of Medicine, Gulu University, Gulu, Uganda

**Keywords:** Mucinous adenocarcinoma of the colon, laparotomy, hepatic flexure, ultrasonography, hereditary non polyposis colorectal carcinoma (HNPCC)

## Abstract

We present a histologically proven mucinous adenocarcinoma of the colon in a 40 year old female from Gulu, Northern Uganda. Her elder sister died at 25 years with advanced adenocarcinoma of colon similarly with her mother who died of the same illness 10 years apart. Using the Amsterdam criteria for the diagnosis of the carcinoma of the colon, this is descriptive of Hereditary Non Polyposis Colorectal Carcinoma (HNPCC). Blood examinations revealed microcytic hypochromic anaemia. The Renal and Liver function parameters were essentially normal. The abdominal ultrasonography showed an ill-defined mass in the right hypochondrial region which was heterogeneous with central echogenicity approximately 7.2cm wide and with no intra-abdominal lymphadenopathy or ascitis. At laparotomy, the sonographic findings were confirmed with a demonstrable mass in the hepatic flexure of the colon with hyperemic areas on its serosa. Macroscopically, there was an annular fungating mass with a central necrosis in the hepatic flexure measuring over 7.0cm. Histology of the colonic tumour showed a mucinous adenocarcinoma of the colon (Duke's B). This finding highlights the occurrence of colonic adenocarcinoma in the young person in Northern Uganda, a finding which draws the attention of the medical community towards having a higher index of suspicion for carcinoma of the colon in patients with similar presentation.

## Introduction

There is paucity of literature on the incidence and prevalence of carcinoma of the colon in Northern Uganda. However, according to the Kyadondo Cancer Registry (KCR) in Kampala; its incidence in Uganda is 9.0% [1] (Table 1) and 7.6% in Kenya [2]. The reason for the high prevalence and incidence of carcinoma of the colon in Uganda compared to the other East African countries is not well understood. More so, in Northern Uganda, it presents commonly among the older age group which gives challenges to clinicians in getting timely diagnosis for the young ones. It is observed that diagnostic difficulties and inappropriate treatment may happen due to the variable clinical appearances of the illness. In the western countries, familial cancer of the colon is the second commonest type (25-30%) of carcinoma of the colon after the sporadic type [3]. The commonly hereditary colonic carcinoma (Familial Adenomatosis polyposis (FAP) and Hereditary Non-polyposis colorectal carcinoma (HNPCC))° which are autosomal dominantly inherited disease account for <1% and 3-5% of all colonic carcinoma respectively [3]. We present a case of a 40 year old female who was successfully managed at Gulu Regional Referral Hospital, one of the teaching Hospitals of Gulu University, Gulu, Uganda. This case report is also presented in remembrance of one of the senior doctors who recently died of colorectal carcinoma in Uganda at the age of 59 years (Dr. MM).

**Table 1:** Age standardized incidence rates (per 100,000) in 3 time period and average annual % change in Age-standardized rates in the period 1991-2006 *(Donald Maxwel Parkin et al (2009). Changing cancer incidence in Kampala, Uganda, 1991–2006)*

Year	1991-1995	1996-2001	2002-2006	Annual % change (95% ConfidenceInterval)
Incidences of carcinoma of the colon				
Male	7.80%	7.30%	7.50%	0.5%(-3.3, 4.2)
Female	5.20%	8.10%	9.00%	6.3%(2.3, 10.3)

## Patient and observation

A 40 year old female, a wife to a senior Medical Doctor in Northern Uganda presented to Gulu Regional Referral Hospital Surgical Out-patient Department (SOPD) with a dull aching abdominal pain in the right hypochondrial region which had lasted 7 months and an abdominal mass in the same area for 5 months. The abdominal pain was not colicky in nature but progressive and radiated to epigastric region and relieved only temporarily and partially by some analgesics. She reported passage of dark colored stool and associated progressive weight loss over the period. There was no reported history of vomiting, diarrhoea or passage of mucoid stool and neither were these symptoms associated with abdominal distension, vomiting or early morning diarrhoea but associated with flatulence which she described as of “rotting smell ”. She reported a history that her mother and elder sister passed on due to a similar illness but for the elder sister, she died when she was 25 years old having been diagnosed with an advanced adenocarcinoma of the colon at one of the hospitals in Northern Uganda. Her mother too died due to a similar condition but at the time of her death she was middle aged. She denies any history of smoking cigarettes or drinking alcohol in her entire life but reports that they normally used firewood for cooking since childhood. She had moved to several health facilities in Gulu seeking medical treatment and she was being treated for Peptic Ulcer Disease (PUD) for which she only got mild relieve from the pain and discomfort. At the time of her admission she had a booklet of medical forms which when combined could be measured at about 10cm in thickness. In all, the clinicians continued to diagnose and treat her with Peptic Ulcer Disease (PUD) and continued to prescribe antacids and other proton pump inhibitors for her treatment. The mass was even biopsied under ultrasound guided biopsy in one of the major hospitals in the region for which the histology results showed a non-specific inflammation. On general examination, she was a young woman in fair general condition, moderately wasted, afebrile to touch with an axillary temperature of 36^o^C. She had moderate pallor but not jaundiced, she had moderate dehydration, but with no palpable lymphadenopathy.

The abdomen was of normal fullness, moving with respiration. It was soft with mild tenderness in the right hypochondrial and lumbar regions. The liver and spleen were not enlarged but there was an ill-defined mass which was palpable in the right hypochondrial and lumbar regions, which was firm in consistency, nodular, non-tender, relatively mobile, intra-abdominal and with a dull percussion note. There were no other masses palpable and no collateral findings on the abdominal wall. There was no renal or suprapubic tenderness and the spleen and the liver were not enlarged. There were normal findings in both the vaginal and rectal examinations with normal anal tone and the rectum was full with faecal matter. The stool on the examination finger was mucoid stained and smelled like rotten meat. A barium enema was requested but was not done due to socio-economic reasons. A complete blood picture revealed a hypochromic microcytic anaemia. The other laboratory results such as the liver function parameters (serum albumin, serum bilirubin, AST, ALT and other enzymes) and renal function parameters (serum electrolytes [K+, Na+, Cl-, -HC0_3_], serum creatinine, blood urea and nitrogen levels) were all within normal ranges (Table 2). The Abdominal ultrasound showed an ill-defined mass in the right hypochondrial region which was heterogeneous with a central echogenicity approximately 7.2cm wide and with no intra-abdominal lymphadenopathy or ascitis. Ultrasound guided biopsy was conducted but was not conclusive. The histological finding showed a fibro-fatty tissue with chronic inflammation containing some eosinophils. These findings created more dilemma and frustration among the family members that they requested that the patient be transferred to Gulu Regional Referral Hospital for further management.

**Table 2:** The haematological, biochemical and clinical chemistry result of the patient

s/no	Laboratory variables	Values	Normal Ranges
1	RBC	4.26x10^6^/uL	3.80- 6.00x10^6^/uL
2	Hb	9.0g/dL	11.5-17.5g/dL
3	HCT	28.80%	37.0- 52.0%
4	MCV	67.6fL	80.0-98.0fL
5	MCH	21.1pg	27.0-32.0pg
6	MCHC	31.3g/dL	31.0-37.0g/dL
7	Platelet counts	295x10^3^/uL	150-400x10^3^/uL
8	WBC	4.62x10^3^/uL	3.50-11.00x10^3^/uL
9	Neutrophils	2.35x10^3^/uL (50.9%)	1.40-6.00x10^3^/uL
10	Lymphocytes	1.83x10^3^/uL (39.6%)	1.00-4.00x10^3^/uL
11	Monocytes	0.34x10^3^/uL (7.4%)	0.20-0.80x10^3^/uL
12	Eosinophils	0.09x10^3^/uL (1.9%)	0.00-0.40x10^3^/uL
13	Basophils	0.01x10^3^/uL (0.2%)	0.00-0.10x10^3^/uL
14	Serum creatinine level	0.4mg/dl	0.4-1.1mg/dl
15	Blood urea nitrogen level	29.0mg/dl	10.0-50.0mg/dl
16	K+ level	3.7mmol/L	3.5-4.5mmol/L
17	Na+ level	135mmol/L	135-145mmol/L
18	Cl- level	100mmol/L	98-106mmol/L
19	HC0_3_- level	21mmol/L	20.0-26.0mmol/L
20	Serum albumin level	39.0mg/dl	38.0-51.0mg/dl
21	AST	37.0u/L	34.0-78.0u/L
22	ALT	29.0u/L	20.0-57.0u/L
23	Bleeding time	3.15'	
24	Clotting time	5.21'	
25	Blood Group	AB +	

**Follow-up Blood tests:** red blood cells (Thin film): Microcytic hypochromic anaemia with plenty of blast cells and shift to the left; Platelets – they were reduced in numbers (190x10^3^/uL)

**Figure 1:**
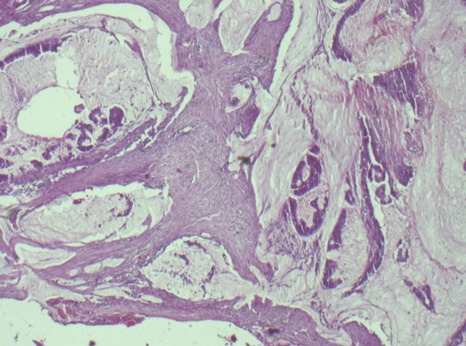
Mucinous adenocarcinoma of the colon at x40 magnification

On the day of admission to Gulu Regional Referral Hospital, she was reviewed by a senior surgeon who took note of the nature of the mass and the positive familial history of carcinoma of the colon and recommended the patient for an exploratory laparotomy since she could not meet most of the costs of investigations requested. The patient was successfully managed surgically at Gulu Regional Referral Hospital main operating theatre. At the Laparotomy, they found a nodular, firm, mobile mass in the hepatic flexure extending 3cm into the transverse colon and 4cm down into the ascending colon not fixed to the posterior wall. A right hemicolectomy was conducted and an end to side anastomosis was performed between the distal ileum and the mid third of the transverse colon. Macroscopically, there was a colonic tumour mass resected was clinically Duke's B classification and there was no gross evidence of mesenteric or mesocolonic lymphadenopathy or ascitis. The liver surface was smooth with no gross evidence of any metastatic sites. The abdomen was then closed in layers. The abdominal stitches were removed in the 10^th^ postoperative day when the wound had healed completely. Gross examination of the resected tumour observed a firm, nodular, annular, fungating, necrotic tumour of the hepatic flexure of the colon measuring 7.0cm in the longest diameter and had not spread beyond the serosa of the colon. Histology of the biopsy taken showed a well differentiated adenocarcinoma of the colon of mucinous type and the margin of the resected transverse colon and the distal ileum were histologically tumour free (Figure 1). The patient did very well postoperatively and was discharged on the 6^th^ postoperative day. She returned to the SOPD on the 10^th^ postoperative day for removal of stitches. She was advised to continue to attend SOPD and also make an effort to visit the Uganda Cancer Institute for further expert advice and treatment from an oncologist. Three weeks later the patient was reexamined and a thorough evaluation of the physical and clinical parameters for any abnormalities was conducted. All parameters were still within their normal ranges. A review of the cancer registry at Lacor Hospital showed that there have been four female patients dignosed with adenocarcinoma of the colon for the period 2013-2015 but all were above 40 years of age (Table 3).

**Table 3:** Shows the Lacor Cancer Registry (LCR) incidence report for adenocarcinoma of the colon in females in the period (2013-2015)

Patient	Age (yrs)	Sex	Address	Histological type
A	50	F	Bwangatira, Gulu	Mucinous adenocarcinoma of the colon
B	43	F	Layibi, Gulu Municipality	Mucinous adenocarcinoma of the colon
C	70	F	Lamogi, Lacor, Amuru	Mucinous adenocarcinoma of the colon
D	46	F	Lamogi, Kilak, Amuru	Mucinous adenocarcinoma of the colon

## Discussion

Colorectal carcinoma is the third commonest malignancy in the developed world after lung and breast cancers [3]. It is reported that the 5 year survival rate from large bowel carcinoma is only approximately 50% even in the developed world in spite of improvements in diagnosis, surgical techniques, preoperative care and postoperative therapies [3]. The incidence of mortality from this disease has little changed over the last 40 years [3]. The diagnostic utility of symptoms predictive of cancer of the colon remains unclear and there is no evidence that delays measured in weeks in diagnosing colorectal carcinoma have any impact on the staging and outcomes of the disease [3]. Patients with high risk of developing colorectal carcinoma include those with known genetic predisposition including strong family history and those with predisposing conditions such as high risk adenomas, long standing ulcerative colitis or previous Colorectal Carcinoma (CRC) [3, 4]. It is reported that over 90% of all the CRC are sporadic and majority of these have strong family history of the disease and are probably genetic in nature [3, 4]. There are a group of risk factors called the Hereditary Non Polyposis Colorectal Carcinoma (HNPCC) and family clusters with no recognizable gene disorder [3]. They are diagnosed clinically using the Amsterdam criteria. The criteria include the following: (a) At least 3 relatives with CRC (b) One should be the first degree relative of the other two; (c) At least two consecutive generations affected (d) One case must be diagnosed before the age of 50 years (e) FAP must have been excluded. The patient we present in this case report had a 25 year old sister who died of the illness and the mother too. At the laparotomy, there was no single polyp observed in the bowel resected. The histological finding from the biopsy was mucinous adenocarcinoma of the colon (Figure 1).

In addition, adenocarcinoma of the colon is reported to be the most common histopathological type of colorectal carcinoma which is ranked fourth in men and third in women in Western Europe and the United States and overall accounts for 98% of cancers of the large intestine [5]. This cancer is reported mainly in association with a western lifestyle (obesity, lack of physical activity, consumption of diets low in fruits and vegetables and over consumption of red meat), hence its predominance in affluent societies [5]. In addition, pre-malignant conditions such as familial adenomatous polyposis coli syndrome and inflammatory bowel diseases have been reported as important associated factors [5]. In sub-Saharan Africa, evidence shows that the incidence of adenocarcinoma of the colon is rising and this has been attributed to changes in lifestyle probably as a result of globalization [4, 5]. In Uganda, the Kampala Cancer Registry which covers a population of about two million people in Kyadondo County, has shown an increased incidence of colorectal carcinoma in the population in the past three decades [4, 5] (Table 1). In the period from 1991 to 2006, the incidences have increased from 5.2 per 100,000 to 9.0 per 100,000 in women [4]. This trend has similarly been observed in other low-income countries where the incidence was once reported to be low [4]. The rising incidence of this cancer is not yet well understood and this calls for a more in-depth study to unveil the factors behind this trend. Cancer control in Uganda, as elsewhere in sub-Saharan Africa, involves meeting the challenges of emerging cancers associated with westernization of lifestyles (large bowel, breast and prostate); although the incidence of cancers associated with poverty and infection (liver, cervix, esophagus) shows little decline, the residual burden of the AIDS-associated cancers remains a major burden [4]. Considering the occurrence of this colonic carcinoma in this young woman in her early years of life, especially when her close relatives have all died of the disease, we propose that there should be a higher index of suspicion when dealing with young people with abdominal symptoms such as presented by this patient in the case report.

## Conclusion

The above patient had adenocarcinoma (Duke's B) of the hepatic flexure of the colon. His elder sister died at 25 years with advanced carcinoma of the colon and the mother too died of carcinoma of the colon. According to the Amsterdam criteria, hereditary non polyposis colorectal carcinoma (HNPCC) is the diagnosis. The need to educate health workers about the increasing incidence and prevalence of colorectal carcinoma calls for higher index of suspicion whenever they are confronted with another of such a patient.

## Competing interests

The authors declare no competing interests.

## References

[1] Wabinga Henry Rocky, Nambooze Sarah, Amulen Phoebe Mary, Okello Catherine, Mbus Louise, Parkin Donald Maxwell (2014). Trends in the incidence of cancer in Kampala, Uganda 1991-2010. Int J Cancer.

[2] Korir Anne, Okerosi Nathan, Ronoh Victor, Mutuma Geoffrey, Parkin Maxwel (2015). Incidence of cancer in Nairobi, Kenya (2004-2008). Int J Cancer.

[3] Acheson Austin G, Scholefield John H (2002). What is new in colorectal cancer. Surgery (Oxford).

[4] Parkin Donald Maxwel, Nambooze Sarah, Wabwire-Mangen Fred, Wabinga Henry Rocky (2010). Changing cancer incidence in Kampala, Uganda, 1991-2006. Int J Cancer.

[5] Tumwine Lynnette K, Kagimu Magid, Ocama Ponsiano, Segamwenge Innocent, Masiira-Mukasa Noah, Wamala Dan (2012). Atypical Presentation of Colon Adenocarcinoma. J Med Case Reports.

